# Metabolic and Immune System Dysregulation: Unraveling the Connections between Alzheimer’s Disease, Diabetes, Inflammatory Bowel Diseases, and Rheumatoid Arthritis

**DOI:** 10.3390/jcm13175057

**Published:** 2024-08-26

**Authors:** Julia Doroszkiewicz, Jan Mroczko, Izabela Winkel, Barbara Mroczko

**Affiliations:** 1 Department of Neurodegeneration Diagnostics, Medical University of Bialystok, 15-269 Bialystok, Poland; mjanek2003@gmail.com (J.M.); barbara.mroczko@umb.edu.pl (B.M.); 2 Dementia Disorders Centre, Medical University of Wroclaw, 50-425 Scinawa, Poland; i.winkel@me.com; 3 Department of Biochemical Diagnostics, Medical University of Bialystok, 15-269 Bialystok, Poland

**Keywords:** Alzheimer’s disease, diabetes mellitus, inflammatory bowel diseases, rheumatoid arthritis

## Abstract

Alzheimer’s disease (AD), diabetes mellitus (DM), inflammatory bowel diseases (IBD), and rheumatoid arthritis (RA) are chronic conditions affecting millions globally. Despite differing clinical symptoms, these diseases share pathophysiological mechanisms involving metabolic and immune system dysregulation. This paper examines the intricate connections between these disorders, focusing on shared pathways such as insulin resistance, lipid metabolism dysregulation, oxidative stress, and chronic inflammation. An important aspect is the role of amyloid-beta plaques and tau protein tangles, which are hallmark features of AD. These protein aggregates are influenced by metabolic dysfunction and inflammatory processes similar to those seen in DM, RA, and IBD. This manuscript explores how amyloid and tau pathologies may be exacerbated by shared metabolic and immune dysfunction. Additionally, this work discusses the gut–brain axis and the influence of gut microbiota in mediating disease interactions. Understanding these commonalities opens new avenues for multi-targeted therapeutic approaches that address the root causes rather than merely the symptoms of these conditions. This integrative perspective could lead to more effective interventions and improved patient outcomes, emphasizing the importance of a unified approach in managing these interconnected diseases.

## 1. Introduction

About 60%–70% of occurrences of dementia in older adults are caused by Alzheimer’s disease (AD). Though substantial progress has been made in understanding genetic and environmental risk factors, as well as the pathology findings connected to this neurodegenerative illness, the exact cause of AD is still unknown. The aggregation and deposition of amyloid-β (Aβ) peptides on the extracellular surface of neuronal cells, which results in the production of Aβ oligomers and fibrils in the brain, is one of the primary characteristics of AD. Additionally, tau protein hyperphosphorylation in AD has an influence on the brain, building up in neuronal microtubules and creating neurofibrillary tangles. These occurrences encourage cytotoxic effects on neural cells that lead to a deterioration in cognition. Another hallmark gaining interest is neuroinflammation occurring during the course of the disease. Neuroinflammation is facilitated by microglia and astrocytes, as well as some immune cells from the periphery that penetrate the brain [1,2]. Even though neuroinflammatory processes at the beginning play significant roles in neuroprotection, chronic and increasing neuroinflammation can negatively impact brain function and cause neurodegeneration and neurological disability [3]. Both innate and adaptive immune responses to infections require microglial cells. Furthermore, it has been demonstrated that the type of microglia plays a critical role in controlling myelination and neurogenesis [1]. Microglial cells become activated and secrete different inflammatory substances under pathological situations. Similar to this, proinflammatory cytokines, pathogenic Aβ, and tau species can stimulate astrocytes, causing them to release these substances [4,5]. Chronically activated microglia and astrocytes can induce brain damage by promoting the formation of toxic protein aggregates and increasing the susceptibility of neurons to cell death through the continuous release of pro-inflammatory cytokines [6,7].

The “glymphatic” pathway facilitates the exchange of interstitial fluid (ISF) and para-arterial cerebrospinal fluid (CSF) in the brain parenchyma, which helps the central nervous system (CNS) clean itself of a variety of hazardous waste products, including amyloid beta [8]. In the elderly brain, neuroinflammation significantly disrupts this system [9,10], which may cause amyloid beta buildup and subsequent neuronal damage. Aquaporin 4 (AQP4) channels, which are highly expressed on the astrocytic endfeet abutting cerebral capillaries, are necessary for lymphatic clearance [11,12]. However, AQP4 polarization is reduced by reactive astrogliosis in response to inflammatory signaling. Furthermore, the aged brain exhibits a substantial upregulation of reactive astrocytes of the “A1” phenotype, which prominently expresses classical complement cascade genes and contributes to neuronal death in many age-associated neurodegenerative disorders [13,14,15].

Alzheimer’s Disease, a neurodegenerative disorder leading to progressive cognitive decline, has traditionally been viewed through the lens of neuropathology. However, growing evidence suggests that metabolic abnormalities, such as insulin resistance and dyslipidemia, play critical roles in its pathogenesis. Similarly, diabetes (DM), characterized by chronic hyperglycemia and metabolic instability, is now understood to have profound effects on the nervous system, potentially accelerating neurodegenerative processes. Rheumatoid arthritis (RA), an autoimmune condition that primarily targets synovial joints, shares common inflammatory pathways with AD, suggesting that chronic systemic inflammation may be a crucial link between these diseases. The inflammation observed in RA is not confined to the joints but can have widespread effects, implicating immune system dysregulation as a shared factor. Moreover, irritable bowel diseases (IBD), encompassing Crohn’s disease and ulcerative colitis, involve chronic inflammation of the gastrointestinal tract. These conditions also demonstrate significant overlap with both metabolic and immune dysfunctions seen in AD, DM, and RA. The gut–brain axis and gut–immune interactions highlight a complex network of bidirectional communication that can influence disease progression and manifestation across these conditions.

This paper aims to explore the converging metabolic and immunological pathways that link Alzheimer’s disease, diabetes, rheumatoid arthritis, and inflammatory bowel diseases. By unraveling these connections, we hope to uncover novel insights into their shared etiologies and identify potential therapeutic targets that could benefit multiple chronic diseases simultaneously. Understanding these commonalities may pave the way for innovative, holistic approaches to treatment and prevention, ultimately improving patient outcomes across these diverse yet interconnected disorders.

## 2. AD and Diabetes

**Figure 1:**
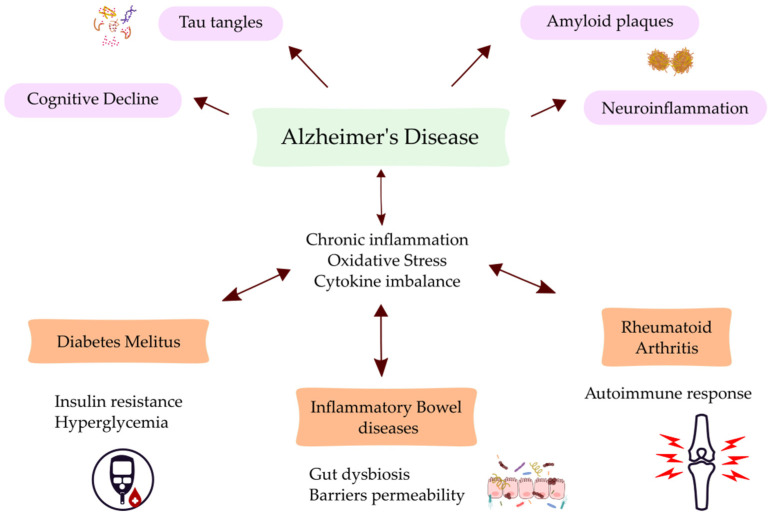
Common aspects of the diseases with additional aspects specific to each disease.

Over the past few decades, there has been a rise in the prevalence of diabetes mellitus (DM). Insulin resistance is a defining feature of type 2 diabetes mellitus (T2DM), which accounts for over 90% of all occurrences of diabetes worldwide, according to the International Diabetes Federation [16]. One in ten persons worldwide have diabetes. It was discovered that T2DM is associated with an increased risk of dementia, similar to dementia itself; however, dementia is also associated with a higher risk of this metabolic abnormality [17]. While AD is a central nervous system issue and T2DM is predominantly a peripheral disease, both conditions are chronic and complex, and they have certain characteristics in common, such as protracted prodromal periods [18]. Furthermore, both demonstrate how inflammation and chronic oxidative stress contribute to the development of the disease. In addition to age, sedentary lifestyle, poor dietary habits, obesity, chronic stress, and genetics are common risk factors [17]. A meta-analysis of DM cases and the risk of all types of dementia, including vascular dementia and AD, revealed a 73% increase in the risk of all types of dementia and a 56% increase in the risk of AD in diabetic patients [19]. The relationship between type 2 diabetes and AD may be explained by a number of theories, including hyperglycemia, which causes glutamate-induced excitotoxicity in neuronal cells and insulin resistance in the brain, which may aid in the buildup of amyloid-β, tau phosphorylation, oxidative stress, the formation of advanced glycation end products (AGEs), and apoptosis [20]. An 11-year follow-up study of a Taiwanese population found that diabetic patients are more susceptible to AD compared to non-diabetic patients [21]. Nevertheless, the connection between AD and T2DM remains unclear. The similarities between AD and diabetes mellitus, as well as the other diseases described in this paper, are shown in Figure 1.

According to MRI studies, hippocampus size has been found to decline in older diabetic individuals. Diabetes (db/db) mice also exhibit age-dependent atrophies of the hippocampus and cortical regions [22]. Reduced neurogenesis and increased neuronal death may have caused these alterations. Throughout adulthood, new neurons are constantly being created in two significant locations of the brain: the subventricular zone and the dentate gyrus of the hippocampus [23]. The growth of neuroprogenitor cells (NPCs) and their eventual differentiation into neurons, astrocytes, and oligodendrocytes is known as neurogenesis. Animal models of obesity and diabetes have been shown to exhibit impaired neurogenesis. For instance, in the Goto-Kakizaki rat, a genetic model of type 2 diabetes, reduced NPC survival has also been noted [24]. In spontaneously obese diabetic mice, a model for type 1 diabetes, the dentate gyrus of newly produced NPCs exhibits a reduction in bromodeoxyuridine-BrdU labeling [25]. In both spontaneously diabetic mice and animals treated with streptozotocin, impaired NPC proliferation is linked to increased glucocorticoid levels and decreased brain-derived neurotrophic factor (BDNF) expression [26]. The stimulation of the NF-kB pathway in the mouse hypothalamus has been linked to the impairment of NPCs caused by a high-fat diet, just like in diabetic rats [27]. According to a recent study, neuronal markers drastically decrease, and NPCs differentiate into a glial phenotype when they are differentiated in the presence of cytokines such as IL-1β, TNF-α, and IL-6 [28]. The results of this study imply that increased levels of circulating cytokines in diabetes may obstruct brain neurogenesis.

Moreover, reduced scores on numerous cognitive function tests are linked to higher levels of glycosylated hemoglobin [29]. Its levels in postmenopausal osteoporotic women without diabetes have been linked to the likelihood of acquiring MCI and dementia [30]. According to the Hisayama study, dementia, from all causes, is linked to glucose intolerance [31]. Individuals with diabetic retinopathy also had low scores on cognitive function tests, according to the Edinburgh type 2 diabetes study [32]. Moreover, higher average blood glucose levels over the previous five years are associated with an increased risk of dementia in both participants with and without diabetes, according to adult changes in the research [33]. In a group of type 2 diabetic individuals, even acute hyperglycemia has been demonstrated to impact mood and cognitive performance [34]. The Maastricht Aging Study found that during a 12-year period, diabetic individuals experienced more cognitive decline than non-diabetic participants, as seen by reductions in word recall and information processing speed [35]. A summary of prospective observational research showed that diabetes raises the risk of cognitive deterioration by a factor of 1.2 to 1.7 [36]. Cognitive performance is correlated with both changes in glycemic control and even the trajectories of glycemic control [37,38]. Because of the risks involved with hypoglycemic episodes, the American Geriatrics Society does not advise reducing the A1c below 8% [39]. Consequently, glucose management must be started early as an interventional technique to stop cognitive impairment [40].

Patients with diabetes may have cognitive deterioration due to a variety of causes. For instance, MRI studies have demonstrated that chronic hyperglycemia-mediated hippocampal impairment and insulin resistance have been linked to AD-like reductions in cerebral glucose metabolism, even in prediabetic people with adequate cognitive function [41,42]. There are numerous insulin receptors located all over the brain. The majority of insulin receptors are found on neurons, where they are part of the postsynaptic density (PSD) and are strongly expressed at the presynaptic axon terminal of synapses. Because of the high concentration of insulin receptors in the entorhinal cortex, frontal cortex, and hippocampal regions of the brain, insulin is thought to be important for learning and memory [43]. Furthermore, improvements in memory following insulin injection have been reported, as well as alterations in insulin receptors in the hippocampal region as a result of spatial learning. Although studies on human and animal models also reported similar effects, it is unclear how specifically insulin affects cognition [44]. Insulin favors long-term depression (LTD) and long-term potentiation (LTP), which may contribute to changes in hippocampal synaptic plasticity, according to the results of animal research. Learning and memory, which are dependent on the hippocampus, are mediated by these chemical pathways [45,46,47]. The expression of the N-methyl-D-aspartate (NMDA) receptor in the membrane is one such mechanism that is controlled by insulin [48]; its expression is governed by the insulin-activated ERK1/2 or PI3-K pathways [49,50]. Additionally, synaptic remodeling—which is necessary for neural plasticity—is influenced by insulin signaling [51]. Further results imply that memory development requires intact insulin signaling [52]. Moreover, the hormone acts on neurons through the AKT and MAPK signaling pathways [53,54]. It is thought to promote neuronal outgrowth, alter catecholamine release and uptake, and control the expression and location of GABA, a key inhibitory neurotransmitter in mammalian brain synapses. It is well-recognized that GABA is involved in sleep, memory, learning, and reproductive system activity [55]. Furthermore, it has a major impact on how the frontal brain regulates neuronal activity, body weight, plasticity, and food intake [56]. Insulin additionally controls the expression of NMDA and α-amino-3-hydroxy-5-methyl-4-isoxazole propionic acid (AMPA) receptors, and it modifies activity-dependent synaptic plasticity through the AKT pathway and NMDA receptor signaling, which results in long-term depression and long-term potentiation [46,57].

Cognitive dysfunction is likely associated with abnormalities in the hypothalamic–pituitary–adrenal axis, inflammatory mediators, rheological variables, and hyperglycemia [58]. An epigenetic mechanism of cognitive impairment in elderly obese individuals with diabetes has been proposed as a result of higher histone deacetylases class IIa in the brains of diabetic people [59]. Insulin resistance causes Akt—a protein involved in several physiological functions, including glucose metabolism—to become less activated. It also inhibits GSK3β, one of the kinases that phosphorylate tau. Consequently, elevated GSK3β activation during insulin resistance may result in tau hyperphosphorylation, which is a crucial element of the neurofibrillary tangles that are seen in AD patients’ brains. Insulin and Aβ peptide are broken down by an enzyme that cleaves insulin (Insulin Degrading Enzyme [IDE]). Consequently, IDE is sequestered from Aβ by hyperinsulinemia, which promotes its accumulation [60,61]. Among the shared pathways found in the brain tissues of AD and diabetes patients are protein misfolding, oxidative stress, and inflammation [40,62]. The accumulation of advanced glycation end products, a significant contributor to diabetic problems, has been discovered in the brain.

In type 2 diabetes, amyloid polypeptide (AP), a protein co-expressed and released with insulin, causes cell death in the islet of Langerhans. In an aquatic environment, AP spontaneously produces amyloid clumps, much like the Aβ protein in AD. The structural characteristics of these proteins showed a significant overlap (90%) according to the alignment analysis. A locally produced amyloid protein is deposited in both disorders, along with a slow reduction in cell count. Among these harmful processes is the chaperone protein pathway, a protein trafficking system that binds developing proteins and helps move them around the cell [63]. Interestingly, patients with AD showed a higher frequency of islet amyloid; nevertheless, brain amyloid did not increase in T2DM patients compared to the normoglycemic group; instead, the density of neuritic plaques was linked to the length of diabetes [63].

The microtubule-associated protein tau, which shares physicochemical characteristics with the condition in the brain, is also found in human islets of Langerhans, despite tauopathies being linked to degenerative conditions such as AD. According to Maj et al., beta-cell-derived mouse models with dramatically reduced insulin transcription, translation, and secretion overexpress the tau protein, highlighting the significance of tau-phosphorylation and dephosphorylation balance for appropriate insulin function [64]. It is interesting to note that pancreatic β cells from AD patients have deposits of the proteins tau and Aβ in their cytoplasm, as reported by Martinez-Valbuena et al. in T2DM patients with normal neuropathological testing [65].

In addition to the metabolism of amyloid precursor protein and the phosphorylation of tau protein, oxidative stress, impaired energetics, mitochondrial dysfunction, inflammation, lipid metabolism, and membrane lipid deregulation are among the numerous biochemical processes that also have an impact on AD and T2DM [66]. Neurotoxicity in AD is linked to mitochondrial failure and the generation of reactive oxygen species, both of which are observed in the condition [67]. It is also known that decreased cerebral glucose uptake develops decades before cognitive loss in AD. As a result, AD may be viewed as a metabolic disease mediated by brain insulin resistance, as numerous studies have demonstrated that insulin resistance might contribute to its pathogenesis [68]. Furthermore, dietary habits, gut microbiota, and peripheral metabolism may have an effect on the metabolic impairment in type 2 diabetes and Alzheimer’s disease [66].

Another protein connected to diabetes, Alpha-2-macroglobulin (A2M), was one of the proteins shown to be elevated in both the AD and T2DM groups relative to the control group in studies [69]. Among the most prevalent proteins in the peripheral blood circulation, A2M is the main non-immunoglobulin molecule. It can induce the transcriptional activation of multiple genes linked to cell proliferation/hypertrophy and atherosclerosis, as well as inhibit a wide range of proteases and pro-inflammatory cytokines [70]. A correlation has been reported between salivary levels of A2M and blood levels of HbA1c, triglycerides, fasting, and postprandial blood sugar in T2DM subjects [71,72]. The relationship between plasma A2M and DM has long been known [73]. According to research by Seddighi et al., there is a significant correlation between plasma A2M levels and the concentrations of total tau and phosphorylated tau in CSF [74]. Additionally, men who are cognitively normal but have higher serum levels of A2M are at a higher risk of developing clinical AD. Due to its ability to promote the aggregation of misfolded Aβ peptide, they postulated that A2M, an acute phase protein and chaperone protein, may be involved in the inflammation and pathogenesis of preclinical AD [74,75]. As a result, α2M appears to be significant in the pathophysiology of both AD and T2DM [69].

Neuroinflammation has been identified as an important factor in the pathophysiology of AD [76,77]. The idea that the central nervous system is immune-privileged has been called into question due to the reciprocal interactions between peripheral and central inflammation [78]. “Inflammaging” is the term used to describe chronic low-grade inflammation that contributes to aging-associated morbidity and mortality [79]. Insulin resistance and the pathophysiology of type 2 diabetes are also significantly influenced by systemic inflammation [80]. In general, microglia respond to peripheral inflammation in an adaptable manner. After exposing mice to IL-1β and TNFα systemically, the hippocampus exhibits the production of cytokines and chemokines [81]. According to a different study, immune system stimulation in mice causes brain pathologies similar to AD, which include the accumulation of APP and its proteolytic fragments as well as changes in Tau phosphorylation [82]. Higher peripheral amounts of IL-6, TNF-α, IL-1β, TGF-β, IL-12, and IL-8 are associated with AD, according to a meta-analysis of forty studies measuring peripheral blood cytokine concentrations [83]. Patients with AD who are in the early stages of the disease have activated peripheral immune cells in circulation [84]. Peripheral inflammation and cognitive dysfunction have also been linked in a number of studies. Peripheral inflammatory indicators, for instance, have been seen in AD and MCI patients [85]. They are correlated with dementia risk, according to meta-analyses of prior research [86]. Increased peripheral TNF-α and IL-1β levels have been linked to an increased risk of AD [87]. When stimulated by phytohemagglutinin (PHA), PBMCs obtained from MCI patients produce higher levels of IL-6 and IL-8 than healthy elderly controls [88]. These proteins are correlated with post-operative cognitive impairment [89]. It has been demonstrated that proinflammatory cytokines can cross the BBB [90,91]. As a result of the aging process, microglia respond to peripheral inflammation more severely and persistently, acting as a priming stimulus for them [92]. Gene expression analysis has demonstrated that brain-specific inflammatory responses are triggered by peripheral inflammation [93]. An increase in central inflammation is also likely to result from harm to the blood–brain barrier and subsequent immune cell infiltration. It is unclear whether cerebral inflammation develops from peripheral inflammation or the other way around. To fully comprehend the molecular connections between diabetes, obesity, and AD in relation to inflammation, more study is required. What is worth noting is the connection between DM and other inflammatory diseases described in our work. Diabetes not only affects the brain but also has an impact on, for example, IBD. A recent meta-analysis showed that by raising the incidence of hospitalization and infections but not of IBD-related complications or mortality, DM may have a deleterious effect on the course of IBD [94].

## 3. AD and Inflammatory Bowel Diseases

The two primary types of inflammatory bowel disease are ulcerative colitis (UC) and Crohn’s disease (CD). These variants can be identified by distinctions in genetic predisposition, risk factors, and clinical, endoscopic, and histological findings, despite certain similar traits. The exact etiology of inflammatory bowel disease is uncertain, although, in genetically predisposed individuals, the response of the mucosa to commensal gut flora appears to be dysregulated, leading to inflammation of the colon [95,96]. In ulcerative colitis, inflammation is typically limited to the mucosal surface. Starting in the rectum, the condition usually spreads proximally and continuously throughout the colon; however, a caecal patch of inflammation may be present in certain patients with proctitis or left-sided colitis. The degree of colonic involvement determines the stratification of disease distribution, which ranges from proctitis to extensive colitis (pancolitis) or left-sided colitis [95,97]. Crohn’s disease involves a complicated interaction of genetic predisposition, environmental variables, and changed gut microbiota that may cause dysregulated innate and adaptive immune responses. In a typical clinical setting, a young patient may present with persistent diarrhea, exhaustion, weight loss, and abdominal pain [98]. It is presented with skip lesions and transmural inflammation that can involve the entire gastrointestinal tract, extending from the mouth to the anus.

Overall, there are some papers that have studied the connection between the probability of AD and IBD. In Zhang et al.’s paper, they showed that patients with IBD were diagnosed with dementia more often than controls. Moreover, the diagnosis was performed at a lower median age than for the control group [99]. This information was also confirmed by Zingel et al. [100]. Similar results were obtained by Sand et al. However, they linked the slightly increased incidence with IBD patients’ more frequent visits to the doctors, resulting in higher detectability of dementia [101]. On the other hand, Huang et al. described that their results, although showing a higher incidence of dementia in IBD patients, are not strong and might be affected by other diseases [102]. Therefore, there is a need to carefully look at the data because there might be a bias connected to other issues.

It is commonly known that IBD is linked to gut microbial dysbiosis. But as of yet, no specific bacterium or microbial environment has been identified as the cause [103,104]. When comparing the intestinal microbiota of IBD patients to that of healthy controls, there are both qualitative and quantitative differences. Short-chain fatty acid (SCFA)-producing bacteria are less prevalent, mucosa-associated bacteria are more prevalent, and total microbial diversity is lower [105,106]. The intestinal barrier’s integrity and the immune system of the host are both impacted by dysbiosis. In people who are genetically vulnerable, this may result in an incorrect mucosal immune response and intestinal inflammation, which could eventually contribute to the onset of IBD. Disorders of the mucosal epithelial barrier that lead to the translocation of gut bacteria stimulate the hyperactivation of the mucosal immune system and the generation of proinflammatory cytokines, which in turn fuel the inflammation that is seen in patients with IBD [103,107]. The gut–brain axis, which is the term for the two-way communication between the gut and the brain, is mostly regulated by gut bacteria, according to numerous recent research. Endocrine, immunological, neurological, and metabolic pathways are only a few of the intricate and mainly unknown pathways that allow gut microorganisms and the brain to interact [108]. Gut hormones carry endocrine messages, while cytokines carry immunological information [109]. The vagus nerve is a vital pathway for the neural link between the gut microbiota and the central nervous system. This communication is thought to be mediated by molecules such as neurotransmitters, microRNA (miRNA), SCFA, and short non-coding RNA (sncRNA) [110]. Gut microbiota may have an impact on all of these signaling pathways; however, it is hypothesized that the pathology of AD is caused by the gut microbiota’s influence on neurological and immunological pathways [109]. Therefore, changes to these two-way communications in the dysbiosis state may also play a role in the pathogenesis of intestinal disorders and CNS disorders, such as the most common neurodegenerative disease, Alzheimer’s disease [111]. In our previous work we have described this connection in the AD [112]. In patients with AD, there are visible changes in the gut microbiota, which contribute to changes in the patients’ brains. The pharmacology and viability of bioactive chemicals are increasingly being studied as a novel and valuable strategy to address a range of neurological degenerative human disorders. Hericium erinaceus has emerged as one of the most intriguing possibilities within the class of mushrooms known as medicinal mushrooms (MMs). Indeed, a number of diseased brain disorders, including Alzheimer’s disease, have been demonstrated to be ameliorated by certain bioactive chemicals that were derived from H. erinaceus. The effects of erinacines on the central nervous system (CNS) have been linked to a significant increase in the generation of neurotrophic factors in a substantial body of in vitro and in vivo preclinical research [113]. Notably, it has been shown that H. erinaceus has neuroprotective benefits via increasing NGF production, which in turn regulates cholinergic neurons and enhances working memory [114,115,116]. Moreover, it was described as a mushroom with the potential to inhibit the activity of AChE, which is another potential hallmark of AD [117]. However, these positive outcomes are also visible in IBD patients. It was shown that H. erinaceus extracts may enhance the host immunity in vivo IBD model and encourage the growth of beneficial gut bacteria, which demonstrates therapeutic potential in alleviating IBD by modulating gut microbiota and the immune system [118]. Therefore, we could postulate that consuming this mushroom could be beneficial to both diseases.

An increasing amount of research has demonstrated that neuroinflammation is one of the primary pathways linking microbiota to AD and that the abnormal gut microbiota-to-CNS route also causes β-amyloid deposition in AD [119,120]. Microglia activation is largely dependent on the gut microbiota, and it has been noted that altering the gut microbiome, especially with regard to bacteria that produce SCFA, may modify neuroimmune activation [119,121]. The fact that the blood–brain barrier and the intestinal wall barrier become more permeable with aging is also significant. Additionally, this may have an impact on how the intestinal microbiota communicates with CNS and expose the latter to more potentially hazardous particles [108,110].

It is well established that dysbiosis of the gut microbiota and systemic inflammation can also impair the intestinal epithelial and blood–brain barriers’ protective qualities, rendering them more permeable and opening the door for peripheral immune cells to enter the brain. The integrity of the BBB is dependent on the proper makeup of the gut microbiota; SCFAs are important metabolites in mediating this impact [122]. Gut microbiota metabolites may enter the central nervous system more easily as a result of disruptions to the blood–brain and intestinal epithelial barriers, as well as microbiome dysbiosis linked to both CD and UC [123]. Massive amounts of lipopolysaccharides (LPSs) and amyloids formed from bacteria may seep from the gastrointestinal tract, build up in the brain and systemic circulation, and so aid in the etiology of Alzheimer’s disease. It has been suggested that LPSs and amyloids could enter the body directly through a damaged barrier and then enter the body indirectly through cytokines produced by LPS and amyloids or other tiny proinflammatory molecules that are typically transited [120,124]. Furthermore, amyloids and LPSs formed from bacteria can intensify the activation of the NF-κB signaling pathway, worsen the leakiness of the gut, and raise the levels of cytokines like IL-17 and IL-22 that are implicated in the inflammatory process of both IBD and AD [106,124]. Recent research has demonstrated that the immune system may be primed, and the immune response to endogenous synthesis of neuronal amyloid in the brain may be enhanced by exposure to bacterial amyloid proteins in the gut. Amyloid proteins generated from the microbiota have the ability to change the structure of proteins (proteinopathy) and increase inflammation in the nervous system, which can cause or worsen brain disease. Amyloid proteins produced from microbiota have the ability to function as prion proteins through molecular mimicry. This process is known as cross-seeding, wherein one amyloidogenic protein (such as tau, Aβ, α-syn, curli, or prion) induces another to take on a pathological β-sheet structure [125,126].

Gut microbiota dysbiosis represents another potential route by which CD and UC may induce the development of AD. It has been shown that bacterial metabolites, particularly SCFA, and the gut microbiota regulate microglial activation continuously. Defects in the maturation, differentiation, and function of microglia may result from an absence of a complex host microbiota [121]. Numerous investigations have demonstrated that dysbiosis of the gut microbiota is linked to both types of IBD [127]. Reduced intestinal microbiota diversity, a decline in SCFA-producing bacteria (particularly *F. prausnitzii*), a drop in bacteria with anti-inflammatory properties, and an increase in pro-inflammatory bacteria are all linked to CD and UC [127]. Similar to IBD, changes in the gut microbiota have also been linked to the onset of AD through immunological, endocrine, and neurological pathways [108]. Both AD and PD patients have altered gut microbiota compositions, with reduced abundances of important SCFA-producing genera such as Butyrivibrio, Eubacterium, Clostridium, and species *F. prausnitzii* [128]. The increased abundance of Escherichia and Shigella and the decreased presence of *F. prausnitzii* and Eubacterium rectale appear to be closely associated with the brain buildup of ß-amyloid in AD [128,129]. Furthermore, compared to healthy controls, AD patients had reduced abundances of anti-inflammatory bacteria such as Eubacterium hallii, Bacillus fragilis, and Bacteroides fragilis and higher abundances of proinflammatory bacteria like Escherichia, Shigella, and Pseudomonas. Proinflammatory cytokine production and amyloid deposition in the brain were linked to the abundance of proinflammatory taxa and the inhibition of anti-inflammatory taxa [128,129].

Another possibility for the co-occurrence of CD and AD in this instance is that patients with both diseases have elevated tau protein expression in common. The pathogenic process in CD is not limited to the lining of the epithelium. All parts of the gastrointestinal wall are affected, nevertheless, including the enteric nervous system (ENS), whose composition and neurochemical characteristics are changed during CD [130]. Prigent et al. showed that patients with CD but not UC have higher submucosal and myenteric plexus levels of tau expression [131]. This elevation is most likely caused by the Nrf2/NDP52 pathway. Like their counterparts in the central nervous system, enteric neurons have also been shown to produce tau protein in other recent investigations [132,133]. This demonstrates that CD and AD have a close association. Remarkably, these results are comparable to those for α-synuclein, which is overexpressed in the ENS in CD [134,135].

Interestingly, Dong et al. discovered that patients with UC and AD share genes *PPARG* and *NOS2.* Knowing that these genes reflect distinct polarization directions in macrophages and microglia, they performed an additional analysis using APP/PS1 and DSS-induced mice to examine any possible connections between the two disorders. They verified a significant decrease in the expression level of Arg1—a biomarker of M2-type polarization—in colon macrophages, hippocampal microglia (DSS-induced mice), and hippocampal microglia (APP/PS1 mice), while a significant increase was observed in the expression level of iNOS, a biomarker of M1-type polarization [136]. A summary of the aspects of IBD and AD is presented in Table 1.

Pro-opiomelanocortin (POMC), a common protein precursor, is the source of the complex and phylogenetically old melanocortin system, which includes α, β, and γ-melanocyte-stimulating hormone (MSH) and adrenocorticotropic hormone (ACTH). Post-translational modification of POMC yields precursor peptides (γ-MSH, ACTH, and β-lipotropins) that can be further changed by acetylation, amidation, and glycosylation to produce active mediators. Specifically, the result of the precursor convertase 2 enzyme’s activity on ACTH, an enzyme substrate, is the α-melanotropin group [137,138]. The processes involved with melanocortins include the regulation of melanogenesis, steroidogenesis, neuromodulation, and the modulation of inflammatory processes. In recent years, the system has come to be recognized as significant in cases of persistent inflammatory illnesses of the digestive tract, such as inflammatory bowel diseases, for which a wealth of data has been gathered using colitis-modeling mice. In fact, details about how this kind of mechanism might mitigate the inflammation caused by colitis and impact the intricate cytokine imbalance in the intestinal milieu damaged by persistent inflammation have surfaced. The central nervous system’s main melanocortin receptor is called MC4R [139]. The brainstem, spinal cord, hypothalamus, and cerebral cortex are where it is primarily expressed. Its distribution is mostly nonoverlapping and extends farther throughout the CNS than MC3R. However, the coexpression of MC3R and MC4R is seen in the posterior hypothalamus nucleus, as well as in the ventral periventricular and premammillary nuclei [140]. Because melanocortins are located in the brainstem, they can reduce the expression of proinflammatory cytokines in endotoxemia, sepsis, and other inflammatory diseases by activating various anti-inflammatory pathways via the efferent vagal pathway [137,141,142]. However, preliminary immunohistochemistry data showed that compared to healthy mucosa, disease-affected CD/UC tracts had significantly greater levels of MC3R expression (together with MC4R expression). Thus, the study demonstrated that these receptors are expressed at the intestinal level in IBD patients and that their expression varies according to the severity of the disease [143]. In fact, MC4R’s function and peripheral localizations are becoming more apparent despite its greater central localization. The expression of MC4R has been demonstrated by Panaro et al. in many gastrointestinal tract segments, including the stomach, small intestine, and descending colon. The mRNA encoding for this receptor was highly expressed in a number of cytotypes, including small intestinal cells positive for gastric inhibitory peptide, glucagon-like peptide 1, and cholecystokinin in mice. Its actions appear to be related to a paracrine suppression of electrolyte secretion, which can be stimulated by administering α-MSH [144]. The MC4R has been the subject of research inside the hippocampal area, particularly in relation to AD. Via the cAMP-PKA signaling pathway, it has been demonstrated that the MC4R controls hippocampus synaptic plasticity in both healthy mice and an AD animal model [145,146]. Subsequent research revealed that in several AD mice models, MC4R activation prevents the illness from progressing [147,148,149].

## 4. AD and Rheumatoid Arthritis

One definition of rheumatoid arthritis is a degenerative condition affecting the skeleton. A systemic inflammatory response that damages articular cartilage and bones is a defining feature of the illness [150]. Immune cells typically have a cascading role in the illness process, engaging subsequent cells and mediators. The immune system’s involvement is linked to the activation of the right cells, which causes the release of inflammatory cytokines and matrix metalloproteinases (MMPs) [151]. In the end, these continuing processes lead to the development of excruciating joint swelling and function impairment [152]. The risk factors for RA are not adequately described. There have been suggestions linking the illness to both external and hereditary factors. The etiology of RA is initially associated with CD4+ lymphocytes. These cells activate monocytes, macrophages, and synovial fibroblasts by identifying antigens in the synovial tissue. The cells listed above release metalloproteinases, which contribute to the deterioration of bone and cartilage. These immune cells produce interleukin (IL)-1, IL-6, and TNF-α, which are responsible for the primary inflammatory response in RA, in addition to their degradative activity. Eventually, synovitis—a thicker and swollen tissue—is the result of the complete cascade process [153].

Rheumatoid factor (RF), anti-cyclic citrullinated peptide (anti-CCP), C-reactive protein (CRP), amyloid A protein, and calgranulin are among the suitable biomarkers that are detected in the serum of RA patients [154]. Since amyloid deposition in soft tissues is a result of amyloidosis of the light chains of transthyretin and immunoglobulins, the existence of amyloid structures is particularly interesting. Orthopedic problems are, in fact, directly caused by the accumulating process [155]. According to one study, amyloid deposits have been seen during orthopedic surgeries, particularly in patients older than 70 [156]. Furthermore, research by Donelly et al. shows that tendon sheath-positive markers of amyloid tissue are present in 10% of men over 50 and women over 60 following biopsy [157]. Further research is required on protein amyloid A (SAA, serum amyloid A), which is produced by liver cells. Increased production of SAA is observed in hepatocytes activated by pro-inflammatory cytokines such as TNF-α, IL-1, and IL-6. A study found that some disorders associated with inflammatory pathology can have an excess of SAA. These illnesses include persistent inflammation, which can be caused by long-term infections such as rheumatoid arthritis, osteomyelitis, and tuberculosis, as well as inflammatory bowel disease, genetic disorders, and solid and hematopoietic neoplasms [158]. It is important to remember that pro-inflammatory cytokines are pleiotropic mediators since they stimulate enhanced angiogenesis, the breakdown of connective tissue binder in RA, and increased amyloid activity [159].

Persistent systemic peripheral inflammation affects the AD-specific neurodegenerative processes. When comparing AD patients to healthy controls, it is evident that inflammatory cytokines such as TNF-α, IL-6, IL-1β, TGF-β, IL-12, and IL-18 are active [160]. Because the over-reactivity of the immune system is a common hallmark of both AD and RA, the cytokines described above and their influence are being researched in the etiology of these disorders. It should be taken into consideration when assessing the positive link between AD and RA that AD is far more common in RA patients than in the general population [161]. In a different study, individuals with rheumatic conditions—particularly RA—were shown to have higher cognitive loss as they aged [162]. Anti-inflammatory medication activity has been used to study the consequences of systemic inflammation. Commonly prescribed medications for RA patients—methotrexate and non-steroidal anti-inflammatory medicines (NSAIDs)—lower the incidence of dementia linked to AD, particularly when taken early in the course of the illness [163,164,165]. However, The Alzheimer’s Disease Anti-inflammatory Prevention Trial Follow-up Study (ADAPT-FS) showed that the results do not lend credence to the idea that among persons with a family history of dementia, celecoxib or naproxen prevent AD [166]. It is generally accepted that immune system hyperactivity is the link between these conditions, but more investigation is required to pinpoint the precise relationship between RA and AD. Dysregulation of genes that repress the cell cycle is a common characteristic of both AD and RA, and it also raises the risk of systemic inflammation. Research indicates that age-related alterations in the cell cycle are strongly regulated and that inflammatory changes influence the emergence of pathogenic changes in both illnesses [167].

The existence of coexisting conditions in patients, such as diabetes, peripheral vascular disease, hyperlipidemia, hypertension, and coronary artery disease, was considered in the Chou et al. study [168]. Overall, it was shown that the incidence of AD in individuals with RA was higher than the incidence in individuals without RA. Furthermore, it was demonstrated that among individuals with RA, the relative risk of AD was dramatically elevated by chronic diseases such as diabetes, vascular disease, and coronary artery disease. Interestingly, anti-rheumatic medication therapy has been shown by other research to lower the risk of dementia [169]. Researchers found that cDMARD users had a lower risk of dementia than people who did not take these drugs. Additionally, those on cDMARD had a lower chance of developing dementia following medication use. The usage of MTX (metotrexat) produced the greatest result. Dementia risk was significantly lower after five years of treatment and twice as low at fifteen years. The study’s authors note significant limitations in spite of the findings. Individuals with comorbid conditions such as peripheral vascular disease, interstitial lung disease, anemia, osteoporosis, myocardial infarction, congestive heart failure, and cardiovascular disease were included in the study. Prior to beginning RA treatment, the patients were on statins, analgesics, antihypertensive medications, or proton pump inhibitors [169].

Neurodegenerative abnormalities have an impact on the development of systemic inflammation. The immune system’s activity, which produces inflammatory biomarkers, shows similarities between the pathomechanisms of AD and RA. Numerous bodily functions are mediated by cytokines, which also have an impact on the blood–brain barrier’s integrity and tightness, as well as severely suppress the production of occluding-forming junctions. Additionally, the pathology of amyloid is linked to AD and RA diseases. Aβ plaques in AD make neurons more vulnerable to excitotoxicity, loss of synaptic protein, and cholinergic transmission; in RA, on the other hand, cytokine-stimulated amyloid plays a role in the bone–joint bond’s deterioration. Compared to healthy individuals, RA patients had a much greater incidence of AD. The relationship between the immunological, skeletal, and neurological systems and the organism’s aging processes clarifies the existence of a complex web of interactions. Even though there are some similarities, this mention needs to be handled very carefully. It is possible that the existence of inflammatory markers does not always show a direct cause-and-effect connection between these conditions. It is possible that AD and RA have distinct inflammatory pathways and that the markers listed are the only thing they have in common. To clarify the potential pathways of the immune cascade in both AD and RA and in both diseases concurrently, more research in this area is necessary [170].

## 5. Conclusions

This paper highlights the intricate connections between Alzheimer’s disease, diabetes, rheumatoid arthritis, and inflammatory bowel diseases, emphasizing the shared mechanisms of metabolic and immune system dysregulation. These seemingly distinct conditions are interwoven through complex interactions between metabolic dysfunction, chronic inflammation, and immune dysregulation, providing insights into their shared etiologies. The metabolic pathways involved in insulin resistance, lipid metabolism, and oxidative stress play significant roles in the pathogenesis of these diseases. For instance, insulin resistance, a hallmark of diabetes, has been implicated in Alzheimer’s disease, where it exacerbates amyloid-beta accumulation and tau phosphorylation. Similarly, lipid metabolism dysregulation is common in rheumatoid arthritis and inflammatory bowel diseases, contributing to systemic inflammation and tissue damage. Chronic inflammation, driven by immune system dysregulation, serves as a critical link between these conditions. The inflammatory cascade, characterized by pro-inflammatory cytokines and immune cell activation, is a shared feature of Alzheimer’s disease, rheumatoid arthritis, and inflammatory bowel diseases. This persistent inflammation not only drives disease progression but also contributes to the development of comorbidities, such as cardiovascular disease, which are prevalent across these conditions. Furthermore, the gut–brain axis emerges as a key player in understanding the connections between these diseases. The bidirectional communication between the gut microbiota and the central nervous system influences both metabolic and immune processes. Dysbiosis, or microbial imbalance, is observed in Alzheimer’s disease and the other diseases described in this paper, suggesting that targeting the gut microbiota could be a promising therapeutic approach.

Ultimately, a deeper understanding of the pathophysiological mechanisms shared by these diseases can revolutionize how their treatment and management are approached. By focusing on the commonalities, we can develop holistic, multi-targeted therapies that address the underlying causes rather than just the symptoms of these conditions. This integrative approach is promising for improving patient outcomes and quality of life across a spectrum of chronic diseases, ushering in a new era of personalized and precise medical care. The diseases discussed in the paper involve complex networks of interactions. These include genetic predispositions, environmental triggers, immune responses, and metabolic processes. The challenge lies in disentangling these overlapping factors to identify specific pathways that contribute to each disease. The interactions between inflammation, immune dysregulation, and metabolic changes are not fully understood, making it difficult to establish clear causal links. Additionally, more longitudinal studies are crucial for understanding how metabolic and immune dysregulation contributes to the onset and progression of these diseases. Without long-term data, it is difficult to determine how early metabolic or immune changes influence the development of these conditions. Another aspect is a huge need for biomarker research. However, the complexity of multifactorial interactions, the scarcity of longitudinal studies, difficulties in biomarker identification and validation, and the limitations of animal models pose significant hurdles. Overcoming these challenges requires a multifaceted approach, including the development of more sophisticated research methodologies, greater investment in long-term studies, and enhanced collaboration between basic scientists and clinicians. Only by addressing these limitations can we hope to unravel the intricate connections between these diseases and develop more effective diagnostic and therapeutic strategies.

## Figures and Tables

**Table 1:** Table presenting the comparison of characteristics of inflammatory bowel diseases and Alzheimer’s disease.

Aspect	Ulcerative Colitis	Crohn’s Disease	Connection to AD	Ref.
Primary Features	Inflammation limited to mucosal surface; continuous from rectum proximally	Transmural inflammation; skip lesions; can affect any part of GI tract from mouth to anus	Both UC and CD linked to increased risk of dementia; connection to neuroinflammation and amyloid deposition	[95,96,99,100,101]
Clinical Presentation	Proctitis, left-sided colitis, pancolitis; limited to colon	Persistent diarrhea, weight loss, abdominal pain; can involve entire GI tract	Higher incidence of dementia in IBD patients; possible earlier onset and increased detection due to frequent medical visits	[95,98,99,100,101]
Endoscopic and Histological Findings	Continuous mucosal inflammation, can include caecal patch in some cases	Transmural inflammation with skip lesions; can involve any part of GI tract	Similar histological patterns in neurodegeneration and inflammation seen in both IBD and AD	[95,96,103,104]
Gut Microbiota	Dysbiosis with decreased SCFA-producing bacteria, increased mucosa-associated bacteria	Reduced diversity, decreased SCFA-producing bacteria, increased pro-inflammatory bacteria	Altered gut microbiota linked to AD through immune and inflammatory pathways	[103,104,105,106]
Impact on Gut-Brain Axis	Increased gut permeability; potential impact on CNS due to dysbiosis	Gut–brain axis dysregulation; potential for microbial metabolites to affect CNS	Gut microbiota influence on neuroinflammation and amyloid deposition; increased gut permeability impacts CNS exposure	[107,108,109,110]
Role of SCFA and Inflammatory Cytokines	Lower SCFA-producing bacteria; increased pro-inflammatory cytokines	Altered SCFA production and increased pro-inflammatory cytokines	SCFA and cytokines impact neuroinflammation and potentially AD progression	[103,104,111,112]
Potential for Cross-Seeding of Amyloids	Not well-defined but potential for amyloids to affect neuroinflammation	Evidence of microbial amyloids contributing to proteinopathy and inflammation	Gut-derived amyloids may impact AD pathology through cross-seeding and neuroinflammation	[119,120,121]
Tau Protein Expression	Generally not elevated in UC	Higher tau expression in enteric nervous system (ENS)	Elevated tau in ENS linked to AD; potential shared mechanisms in tau pathology	[123,124,125,126]

## Data Availability

Not applicable.
